# Effect of Comorbidity on Prostate Cancer–Specific Mortality: A Prospective Observational Study

**DOI:** 10.1200/JCO.2016.70.7794

**Published:** 2017-09-20

**Authors:** Prabhakar Rajan, Prasanna Sooriakumaran, Tommy Nyberg, Olof Akre, Stefan Carlsson, Lars Egevad, Gunnar Steineck, N. Peter Wiklund

**Affiliations:** Prabhakar Rajan, Queen Mary University of London; Prabhakar Rajan and Prasanna Sooriakumaran, University College London Hospitals National Health Service Foundation Trust; Prabhakar Rajan, Barts Health National Health Service Trust, London; Prasanna Sooriakumaran, University of Oxford, Oxford; Tommy Nyberg, University of Cambridge, Cambridge, United Kingdom; Tommy Nyberg, Olof Akre, Stefan Carlsson, Lars Egevad, Gunnar Steineck, and N. Peter Wiklund, Karolinska Institutet; and Olof Akre, Stefan Carlsson, and Lars Egevad, Karolinska University Hospital, Stockholm, Sweden.

## Abstract

**Purpose:**

To determine the effect of comorbidity on prostate cancer (PCa)–specific mortality across treatment types.

**Patients and Methods:**

These are the results of a population-based observational study in Sweden from 1998 to 2012 of 118,543 men who were diagnosed with PCa with a median follow-up of 8.3 years (interquartile range, 5.2 to 11.5 years) until death from PCa or other causes. Patients were categorized by patient characteristics (marital status, educational level) and tumor characteristics (serum prostate-specific antigen, tumor grade and clinical stage) and by treatment type (radical prostatectomy, radical radiotherapy, androgen deprivation therapy, and watchful waiting). Data were stratified by Charlson comorbidity index (0, 1, 2, or ≥ 3). Mortality from PCa and other causes and after stabilized inverse probability weighting adjustments for clinical patient and tumor characteristics and treatment type was determined. Kaplan-Meier estimates and Cox proportional hazards regression models were used to calculate hazard ratios.

**Results:**

In the complete unadjusted data set, we observed an effect of increased comorbidity on PCa-specific and other-cause mortality. After adjustments for patient and tumor characteristics, the effect of comorbidity on PCa-specific mortality was lost but maintained for other-cause mortality. After additional adjustment for treatment type, we again failed to observe an effect for comorbidity on PCa-specific mortality, although it was maintained for other-cause mortality.

**Conclusion:**

This large observational study suggests that comorbidity affects other cause–mortality but not PCa-specific– mortality after accounting for patient and tumor characteristics and treatment type. Regardless of radical treatment type (radical prostatectomy or radical radiotherapy), increasing comorbidity does not seem to significantly affect the risk of dying from PCa. Consequently, differences in oncologic outcomes that were observed in population-based comparative effectiveness studies of PCa treatments may not be a result of the varying distribution of comorbidity among treatment groups.

## INTRODUCTION

Prostate cancer (PCa) is one of the most common causes of male deaths from cancer in Europe.^1^ Whereas watchful waiting (WW) is an accepted method of PCa management, the risk of PCa-specific mortality can be diminished by radical treatment of localized tumors with either radical prostatectomy (RP)^2,3^ or radical radiotherapy (RT). For non–organ-confined disease, androgen deprivation therapy (ADT) effectively palliates and prevents PCa-related complications, but without a survival advantage in the absence of combination treatment with RT for locally advanced tumors.^4^

Comorbidities are medical disorders that coexist with, but are distinct from, the primary diagnosis.^5^ As with PCa, comorbidity is also age related and can influence the decision, timing, and modality of treatment selection. Because the survival advantage of radical therapy for PCa is typically observed only 10 years after treatment,^2^ current European guidelines recommend radical treatment with curative intent in patients with a > 10-year life expectancy.^4^

Comorbidity is highly prevalent among patients with cancer^6^ and may adversely affect both competing-cause and cancer-specific mortality,^7,8^ depending on the measures of comorbidity and survival. Evidence for an effect in patients with PCa is conflicting,^1,8,9^ and how comorbidity influences PCa-specific mortality is unclear. Here, by using a population-based observational cohort, we test the hypothesis that PCa-specific mortality is not affected by comorbidity after accounting for patient and tumor characteristics and treatment type.

## PATIENTS AND METHODS

### Study Cohort

Our study cohort is based on the Prostate Cancer Database Sweden (PCBaSe), which is described elsewhere.^10,11^ In brief, PCBaSe is a composite population-based data set that links the National Prostate Cancer Register, the Swedish Cancer Register, the cause of death register, and six other national registers by a unique personal identity number that is assigned to every Swedish resident. PCBaSe captures more than 98% of all cases of PCa in Sweden diagnosed since 1998^12^ with virtually complete data on year of diagnosis, age, clinical (TNM) stage,^13^ Gleason tumor grade,^14^ or WHO^15^ tumor grade, diagnostic serum prostate-specific antigen (PSA) levels, planned primary treatment within 6 months of diagnosis, county of residence, marital status, educational level, socioeconomic status, comorbidity, and cancer-related events during follow-up.^11^ Neoadjuvant ADT with RP was not recorded, but is a rare management strategy; neoadjuvant ADT with RT has been recorded since 2008 only; and information on ADT after WW was not available. We identified a total of 129,389 men who were diagnosed with PCa between January 1998 and December 2012 and who were observed for survival (or death) until December 2014, which is the last date of update for the cause of death register linkage to PCBaSe. Causes of death were ascertained by using the cause of death register, for which the accuracy of the data on PCa has been validated and reported to be 86%.^16^ After exclusion of those patients whose treatment was unknown (n = 5,427), those who had died before treatment (n = 482), or those who were missing one or more tumor covariates (n = 4,937) we included all patients regardless of primary treatment or otherwise (n = 118,543). Median follow-up time for the included cohort was 8.3 years (interquartile range, 5.2 to 11.5 years). This study was approved by the central research ethics committee and the regional ethical review board in Stockholm (EPN Dnr 2012/499-31/4).

### Comorbidity

Comorbidity—described in PCBaSe by the Charlson comorbidity index (CCI)^17^—was estimated from registrations in the Swedish National In-Patient Register and the Swedish Cancer Register that were retrieved from 10 years before, until the date of PCa diagnosis.^9^ Although outpatient diagnoses were not included, the validity of these registers has been demonstrated to be high for medical diagnoses^18^ and most cancer diagnoses.^19^ CCI is a weighted scoring system that estimates the burden of 17 groups of concomitant diseases (Data Supplement) for each patient,^17^ which results in four comorbidity levels that are scored from 0 (no comorbidity) to ≥ 3 (severe comorbidity). CCI has been previously shown to impact treatment choices for PCa and the subsequent outcomes of patients in PCBaSe.^9^

### Statistical Analyses

Clinicopathologic characteristics were reported as medians and interquartile ranges. Study end points were PCa-specific and other-cause survival. Survival time was defined as the interval between the date of PCa diagnosis and the date of death, emigration, or end of follow-up. When considering one cause of death, deaths that were from a competing cause were treated as censoring time points. Overall follow-up time was calculated by using the reverse Kaplan-Meier method.^20^ Patients were categorized by patient factors (marital status, educational level), tumor characteristics (PSA, clinical grade and stage), and treatment type, namely, RP, RT, ADT, and WW. Data were stratified by CCI (0, 1, 2, or ≥ 3) and treatment type as detailed in the tables and figure legends, respectively. The χ2 and Wilcoxon Mann-Whitney tests were used to test for differences in the distributions of patient characteristics between CCI groups. To adjust for any imbalances in the distribution of covariates among groups, we used stabilized inverse probability weighting, a propensity score–based method for which a situation is emulated in which the groups to be compared are made to have similar characteristics at baseline on the basis of preselected adjustment variables.^21^ For adjustments, we used patient and tumor-related clinical characteristics that were available only at the time of treatment decision, namely, age, marital status, educational level, year of diagnosis, tumor grade, clinical stage, and PSA. Adjustment weights were constructed by using multinomial regression wherein continuous covariates were modeled as restricted cubic splines with three knots. Extreme low or high weights were truncated at 0.25 and 4, respectively. PCa-specific and other-cause survival were compared between the actual and emulated groups by using unadjusted and weighted Kaplan-Meier estimates and Cox proportional hazards regression models to calculate hazard ratios. A cause-of-death–specific analysis, rather than competing-risks analysis, was specifically used to determine whether comorbidity affected PCa-specific or other-cause mortality under the hypothetical scenario that no men in the overall cohort or each treatment group (RP, RP, ADT, or WW) would die of causes other than PCa, or from PCa, respectively.^22^ Statistical analyses were performed with R software v.3.1.2^23^ using the multinom function from the nnet package as well as the Survival Analysis and Regression Modeling Strategies packages. All statistical tests were two sided and performed at a 5% significance level.

## RESULTS

Baseline unadjusted patient and tumor characteristics and treatment type (RP, RT, ADT, or WW) stratified by CCI (0, 1, 2, or ≥ 3) are listed in Table 1 (the data after statistical adjustments are listed in the Data Supplement). At diagnosis, patients with greater comorbidity were generally older than those with no comorbidity. Median PSA at the time of diagnosis was also higher in the comorbid group compared with the group with no comorbidity. Consistent with this, there was a higher proportion of low-grade, localized tumors that were identified in the group of patients with no comorbidity compared with the comorbid group. Consequently, the proportion of patients who were treated by radical therapies (RP or RT) was greater in the group of patients with little or no comorbidity than in the more comorbid groups in which a greater proportion of patients were treated with ADT or WW. Of a total of 87,816 patients in CCI group 0, 16,186 patients in CCI group 1, 9,114 patients in CCI group 2, and 5,427 patients in CCI group ≥ 3 (Table 1) there were 15,403, 3,591, 1,895, and 1,134 PCa-related deaths and 15,203, 5,409, 3,683, and 2,849 deaths from other causes, respectively, by the end of the study period.

**Table 1.**
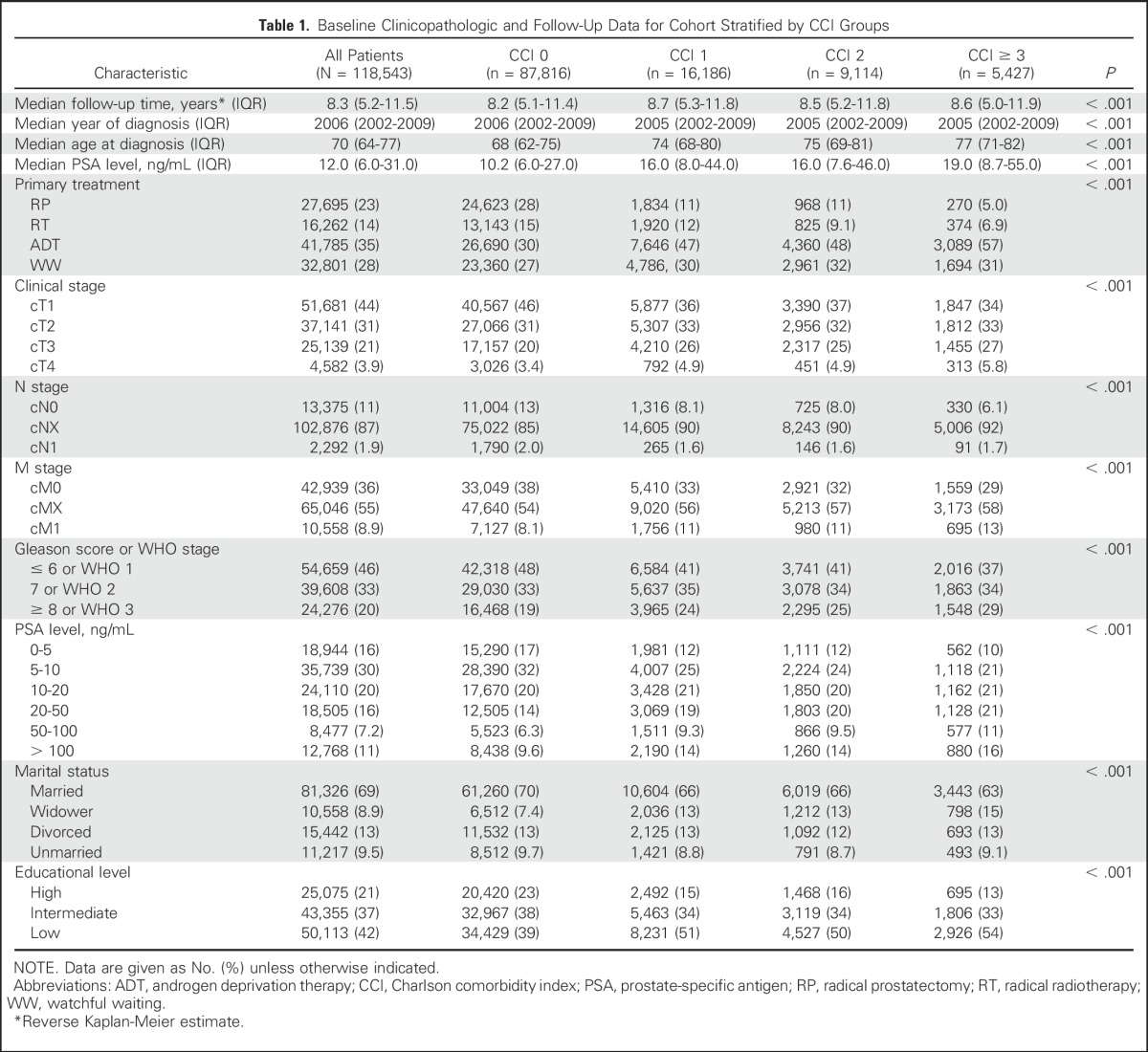
Baseline Clinicopathologic and Follow-Up Data for Cohort Stratified by CCI Groups

**Fig 1.**
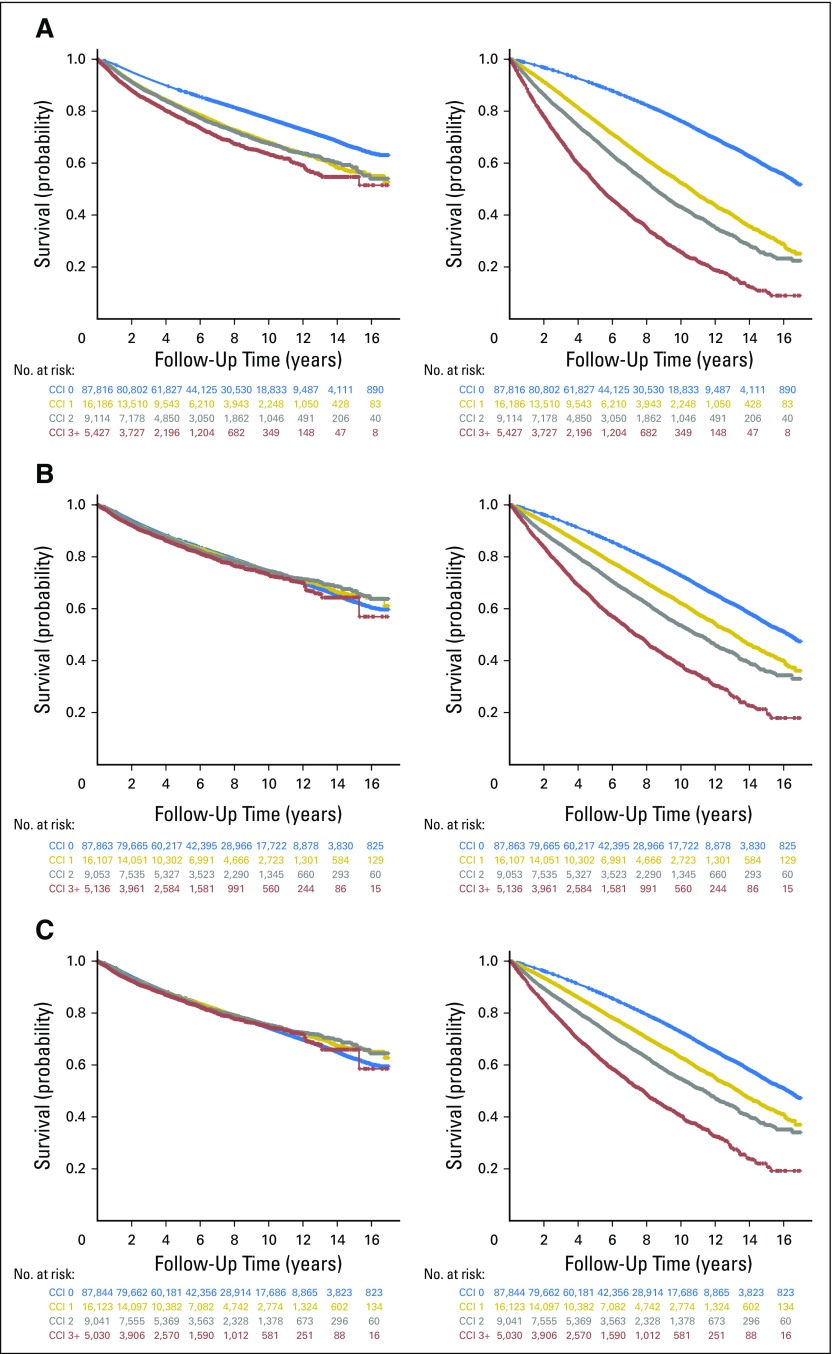
Prostate cancer (PCa)–specific (left panels) and other-cause (right panels) survival for (A) a full cohort of patients without adjustments, (B) a full cohort of patients with adjustments for patient and tumor characteristics, and (C) a full cohort of patients with adjustments for patient and tumor characteristics, and treatment type. Unadjusted and adjusted Kaplan-Meier plots display cumulative survival probability and follow-up time . The number of patients at risk in each Charlson comorbidity index (CCI) group are tabulated at each time point on the *x*-axis with text colors corresponding to the same color for each curve on the Kaplan-Meier plot representing CCI 0 (blue), CCI 1 (gold), CCI 2 (gray), and CCI ≥ 3 (red).

In the complete unadjusted data set, we observed an effect of increased comorbidity on PCa-specific and other-cause mortality, which rose to a 1.99 -fold hazard (95% CI, 1.87 to 2.11) of PCa-specific mortality and a 5.62-fold hazard (95% CI, 5.40 to 5.85) of other-cause mortality for patients with a CCI score of ≥ 3 compared with those with no comorbidity (CCI 0; Table 2 and Fig 1A, left and right panels, respectively). After adjustments for patient and tumor characteristics, the effect of limited comorbidity (CCI score of 1 and 2) on PCa-specific mortality was clearly attenuated and not statistically significant, but was maintained for other-cause mortality across all CCI groups (Table 2 and Fig 1B, left and right panels, respectively). After additional adjustment for treatment type, the association between comorbidity and PCa-specific mortality was again attenuated without clear trends across CCI groups, whereas the effect of increasing comorbidity on other-cause mortality was maintained (Table 2 and Fig 1C, left and right panels, respectively). Of the individual comorbidities that constituted the CCI (Data Supplement), only congestive heart failure and dementia affected PCa-specific mortality after adjusting for patient and tumor characteristics and treatment type (Data Supplement).

**Table 2.**
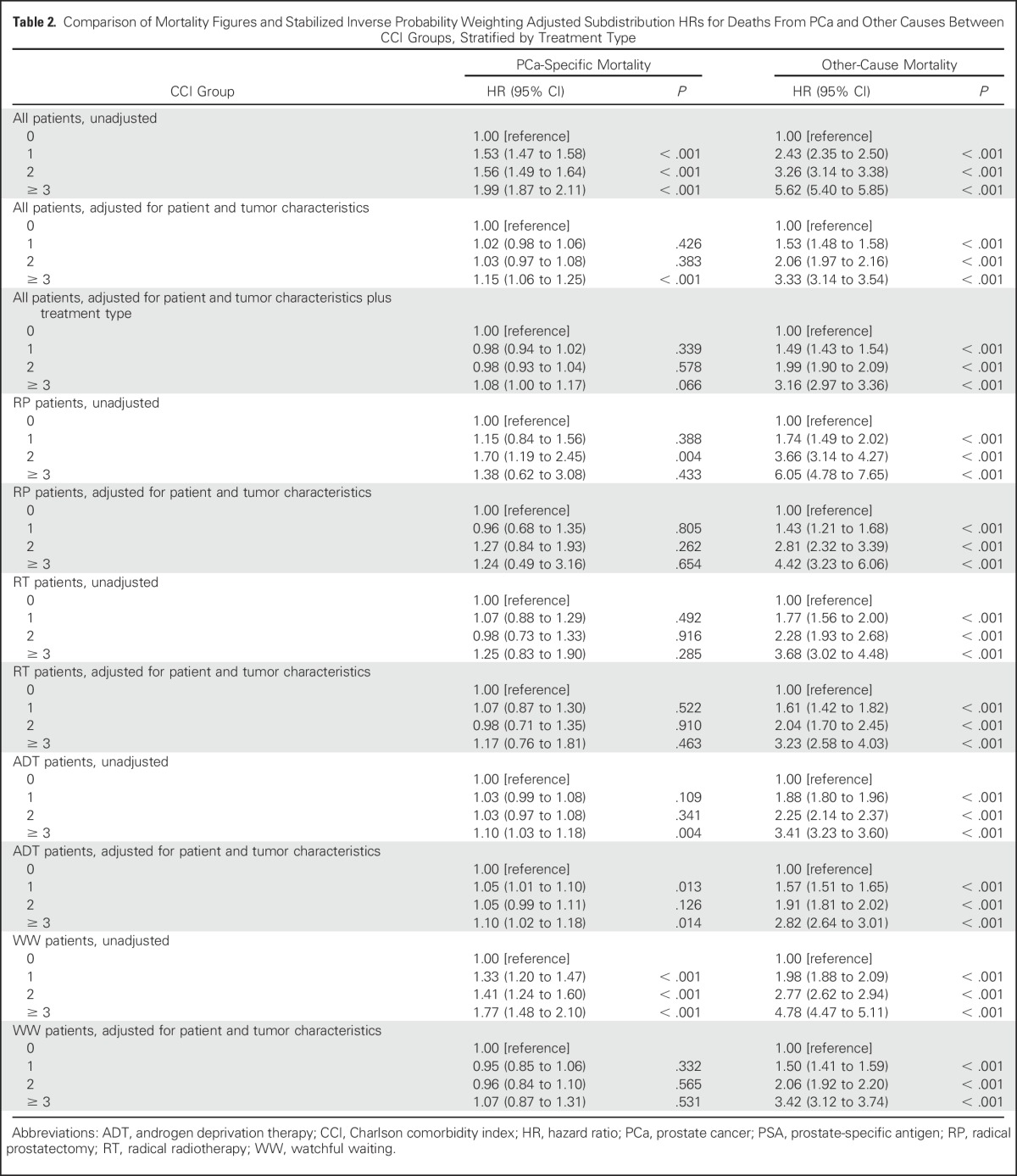
Comparison of Mortality Figures and Stabilized Inverse Probability Weighting Adjusted Subdistribution HRs for Deaths From PCa and Other Causes Between CCI Groups, Stratified by Treatment Type

**Fig 2.**
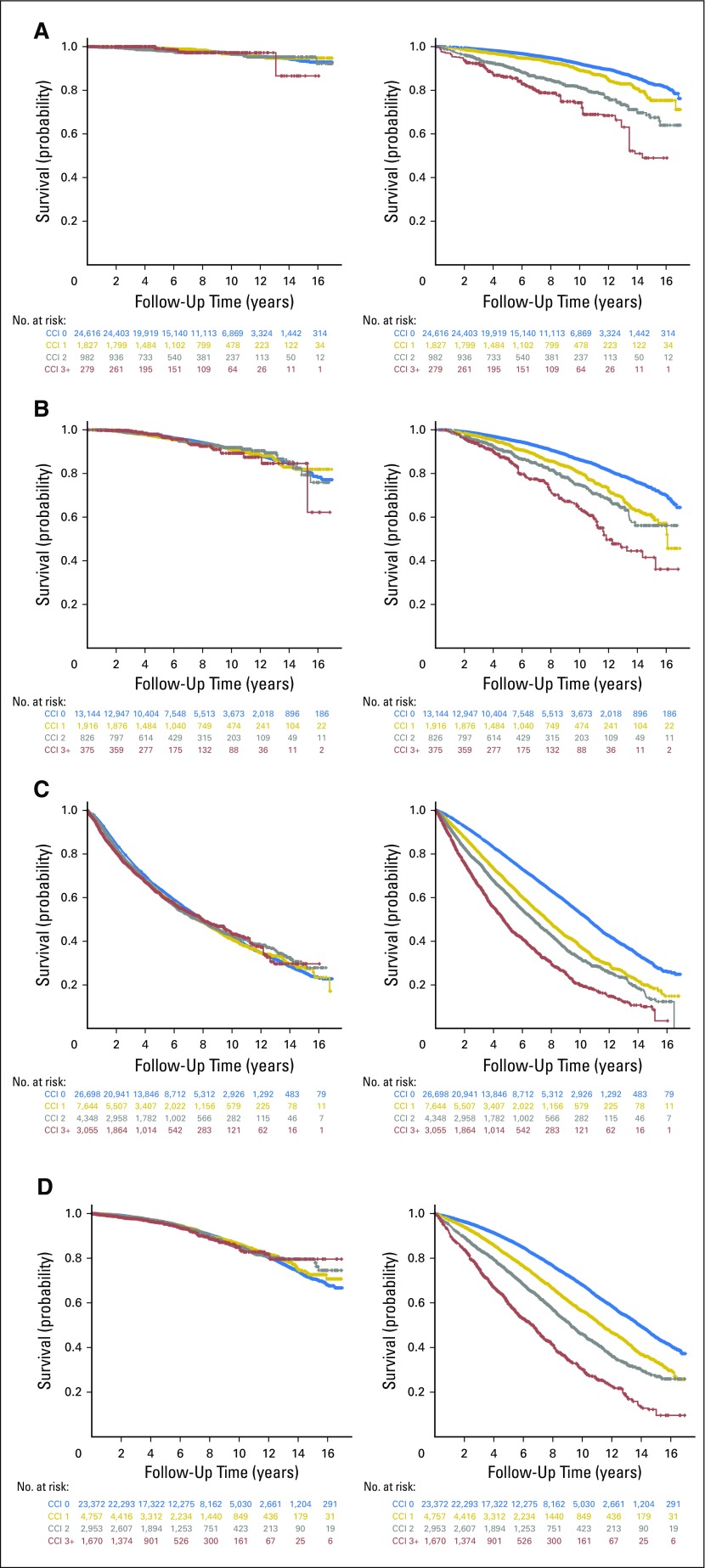
Prostate cancer (PCa)–specific (left panels) and other-cause (right panels) survival for a cohort of patients with adjustments for patient and tumor characteristics, stratified by treatment type. (A) Radical prostatectomy, (B) radical radiotherapy, (C) androgen deprivation therapy, and (D) watchful waiting. Adjusted Kaplan-Meier plots display cumulative survival probability and follow-up time. The number of patients at risk in each Charlson comorbidity index (CCI) group are tabulated at each time point on the *x*-axis with text colors corresponding to the same color for each curve on the Kaplan-Meier plot representing CCI 0 (blue), CCI 1 (gold), CCI 2 (gray), and CCI ≥ 3 (red).

In a subset analysis in which comparisons were repeated separately within each treatment subgroup (Table 2 and Figs 2A-2D, left panels), the only treatment for which an effect of comorbidity on PCa-specific mortality was consistently observed for all CCI groups compared with no comorbidity (CCI score, 0) in unadjusted data was WW, which was lost after adjusting for patient and tumor characteristics (Table 2). For the RP, RT, and ADT subgroups, limited differences in PCa-specific mortality were observed in both unadjusted and adjusted data (Table 2). Additional analyses of a subgroup of patients who were diagnosed and treated between 2008 and 2012 for which information was available on neoadjuvant ADT before RT did not reveal any association between CCI and PCa-specific mortality for patients who were treated with RT (Data Supplement). The most striking effect of comorbidity on other-cause mortality was observed in patients who were treated with RP, where patients had a 4.42-fold hazard (95% CI, 3.23 to 6.06) in other-cause mortality with a CCI score of ≥ 3 (Table 2 and Fig 2A, right panel).

## DISCUSSION

In this large observational study of men with PCa who were treated with RP, RT, ADT, or WW, with a maximum follow-up to 16.99 years, we did not observe a statistically significant effect of comorbidity (as determined by CCI) on PCa-specific mortality after statistical adjustments for patient and tumor characteristics and treatment type; however, we did see an association with a CCI score of ≥ 3 compared with no comorbidity (CCI score, 0) on PCa-specific mortality after adjusting for patient and tumor characteristics alone, which was lost after additional adjustments for treatment type—the reasons for this effect are unclear. Overall, our findings suggest that, after adjusting for patient and tumor characteristics, comorbidity does not seem to significantly impact the risk of dying from PCa after radical treatment (RP or RT) or WW.

Population-based studies that incorporate all disease states^24-28^ and a recent systematic review^29^ suggest that RP may be more effective than RT for the treatment of localized PCa, although a common criticism of these analyses is that the differences observed, at least in part, may be a result of the distribution of comorbidity between treatment groups. The premise here is that lesser comorbidity in the RP group may result in residual bias that accounts for the lower PCa-specific mortality compared with RT; however, considering the lack of an association between comorbidity and PCa-specific mortality for both RP and RT in our present study, differences in oncologic outcomes that have been observed in population-based studies may not be a result of comorbidity-associated bias, although residual confounding is difficult to preclude without random treatment allocation.

To date, the two largest population-based studies have reported similar findings for an effect of comorbidity on other-cause mortality, with discordant findings for an effect on PCa-specific mortality using competing-risk models. A PCBaSe-based study identified an association between increased comorbidity and PCa-specific and all-cause mortality.^9^ A similar effect of comorbidity was identified in all-cause, but not PCa-specific mortality in a study that was based on the Prostate Cancer Outcomes Study population-based cohort from the National Cancer Institute SEER program.^30^ Consistent with this, another large SEER-based study, which excluded all patients who underwent radical treatment, observed similar effects of increased comorbidity on overall survival by using a competing-risks model.^31^ Although the reasons for the differences in findings between the PCBaSe ^9^ and SEER^30^ studies are unclear, possible explanations are the patient self-reporting of comorbidity in the SEER data set^30^ or the misclassification of comorbidity in the PCBaSe data set.^9^ Other smaller studies have demonstrated an association between increased comorbidity and risk of other cause–mortality and all cause–mortality, but not PCa-specific mortality in men with localized PCa who were treated with RP, RT, ADT, or WW.^32-34^ These studies are limited by potential bias associated with the retrospective nature of single-center data and small numbers^34^ or a complete absence of patients who were treated with ADT or WW.^32,33^

The above population-based studies^9,30,31^ that used competing-risk models can help to determine whether the risk of death from PCa differs between groups, allowing certain men to be protected from PCa-specific mortality because of death from other causes. The findings may be relevant in counseling men with comorbidity about radical treatment options for which treatment choices are based on shared decision-making with knowledge of the potential benefits and harms of treatment when considering an individual’s risk of death from PCa and/or other causes. For example, a patient with comorbidity and a greater risk of other-cause mortality compared with PCa-specific mortality may wish to withhold treatment to limit PCa treatment–related morbidity without compromising oncologic outcome. In comparison, our cause-of-death–specific analyses allow us to emulate how comorbidity affects PCa-specific mortality under the hypothetical scenario that no men died of causes other than PCa. We used this method as we wished to determine whether a patient with comorbidity is more or less likely to die of PCa compared with a noncomorbid patient.

Our study has several strengths. First, it utilizes a large, unique composite population-based data set that links nine national registries with complete data collection from the time of diagnosis and during follow-up of more than 98% of men who were diagnosed with PCa in Sweden since 1998, with well-validated patient and clinicopathologic variables. The accuracy of the study end point is high, as the cause of death register in Sweden has been found to be reliable for the correct assignment of cause of death for patients with PCa.^16^ As a result of long-term follow-up, it is possible to identify differences in mortality > 10 years after diagnosis and treatment, thereby increasing the sensitivity of the analyses.

We used stabilized inverse probability weighting propensity score–based statistical adjustments for preselected variables to emulate a situation in which groups to be compared are made to have similar characteristics at baseline; however, unmeasured or misclassified confounders may still bias the estimates. For example, despite the apparent validity of the cause-of-death register,^16^ Swedish National In-Patient Register,^18^ and Swedish Cancer Register, which constitute the CCI score in PCBaSe, it is conceivable that the misclassification of cause of death, or insensitivity of comorbidity estimation, or ability to detect certain conditions may result in bias. In addition, our PCa- and other cause–specific survival analyses may result in bias because of informative censoring if those patients who died early from other causes had at a different risk of death from PCa than did those who survived longer. Hence, similar analyses of large population-based PCa data sets are needed to validate our findings.

Notwithstanding these limitations, our study suggests that comorbidity affects other cause–mortality but not PCa-specific–mortality, accounting for patient and tumor characteristics and treatment type. Regardless of radical treatment type (RP or RT), increased comorbidity does not seem to significantly affect the risk of dying from PCa. Consequently, differences in oncologic outcome that were observed in population-based comparative effectiveness studies of PCa treatments do not seem to be a result of the varying distribution of comorbidity among treatment groups.
